# Incidence and predictors of retreatment in chronic hepatitis B patients after discontinuation of entecavir or tenofovir treatment

**DOI:** 10.1371/journal.pone.0222221

**Published:** 2019-10-04

**Authors:** Te-Ling Ma, Tsung-Hui Hu, Chao-Hung Hung, Jing-Houng Wang, Sheng-Nan Lu, Chien-Hung Chen

**Affiliations:** Division of Hepatogastroenterology, Department of Internal Medicine, Kaohsiung Chang Gung Memorial Hospital and Chang Gung University College of Medicine, Kaohsiung, Taiwan; University of Cincinnati College of Medicine, UNITED STATES

## Abstract

**Background:**

This study investigated the incidence and predictors of retreatment after discontinuation of either entecavir (ETV) or tenofovir disoproxil fumarate (TDF) treatment in Taiwan.

**Methods:**

A total of 535 non-cirrhotic chronic hepatitis B (CHB) patients undergoing either ETV (n = 358) or TDF (n = 177) treatment were enrolled. Patients were followed for at least 12 months after stopping ETV or TDF treatment. Most patients (86.3%) fulfilled the retreatment criteria of Taiwan's National Health Plan.

**Results:**

The 5-year cumulative rates of clinical relapse and retreatment were 52.1% and 47%, respectively, in 160 hepatitis B e antigen (HBeAg)-positive patients, and were 62% and 54.8%, respectively, in 375 HBeAg-negative patients. The median duration from the end of treatment until clinical relapse and retreatment was 40 and 57 weeks, respectively, for all patients. Multivariate Cox regression analysis revealed that discontinuing TDF treatment, old age, male gender, and higher baseline HBsAg levels were independent factors of retreatment in HBeAg-positive patients; old age, HBV genotype B, and higher baseline and end-of-treatment HBsAg levels were independent factors in HBeAg-negative patients. A total of 18.8% of retreated patients satisfied the retreatment criteria of hepatic decompensation according to Taiwan's National Health Plan. Of the 64 patients who had clinical relapse without retreatment, 17 achieved sustained virological remission and 26 did not experience clinical relapse until their last visit after clinical relapse. Four patients developed HBsAg loss.

**Conclusions:**

The 5-year retreatment rate was about 50% in HBeAg-positive and HBeAg-negative patients. Discontinuing TDF treatment was an independent factor of retreatment in HBeAg-positive patients.

## Introduction

To date, hepatitis B virus (HBV) infection still remains one of the most challenging global public health issues—it may cause chronic hepatitis and lead to cirrhosis and hepatocellular carcinoma [1,2]. Entecavir (ETV) and tenofovir disoproxil fumarate (TDF) are potent nucleos(t)ide analogues (NA) that function as first-line therapies for chronic hepatitis B (CHB) [3–5]. However, life-long therapy is usually necessary due to the low rate of hepatitis B surface antigen (HBsAg) loss, and the rate of HBV relapse is typically high after the cessation of NA therapy [6–9].

In our prior study [10], we reported that the 8-year cumulative rates of virological relapse and clinical relapse after stopping either ETV or lamivudine therapy in hepatitis Be antigen (HBeAg)-positive patients were 55.6% and 47.7%, respectively; the corresponding values in HBeAg-negative patients were 69.3% and 58.9%, respectively [10]. However, the rates of retreatment after discontinuation of NA therapy remain unclear. Recent studies have demonstrated that discontinuation of TDF was associated with earlier relapse than the discontinuation of ETV in CHB patients [7,11,12]. Retreatment rates during off-therapy follow-up between ETV and TDF treatment based on the same criteria of retreatment have been rarely compared. However, consensus for retreatment criteria has yet to be determined according to three international guidelines (Asian Pacific Association for the Study of the Liver (APASL), European Association for the Study of the Liver (EASL), and the American Association for the Study of Liver Diseases (AASLD)) [3–5].

A recent study demonstrated that CHB patients with clinical relapse who remained untreated had a 7.34-time higher incidence of HBsAg seroclearance than patients who received retreatment [13]. Additional future studies should focus on clinical outcomes in CHB patients with clinical relapse but without retreatment.

In this study, we investigated the incidence and predictors of retreatment in CHB patients without cirrhosis after the discontinuation of either ETV or TDF treatment.

## Patients and methods

### Patients

**Fig 1:**
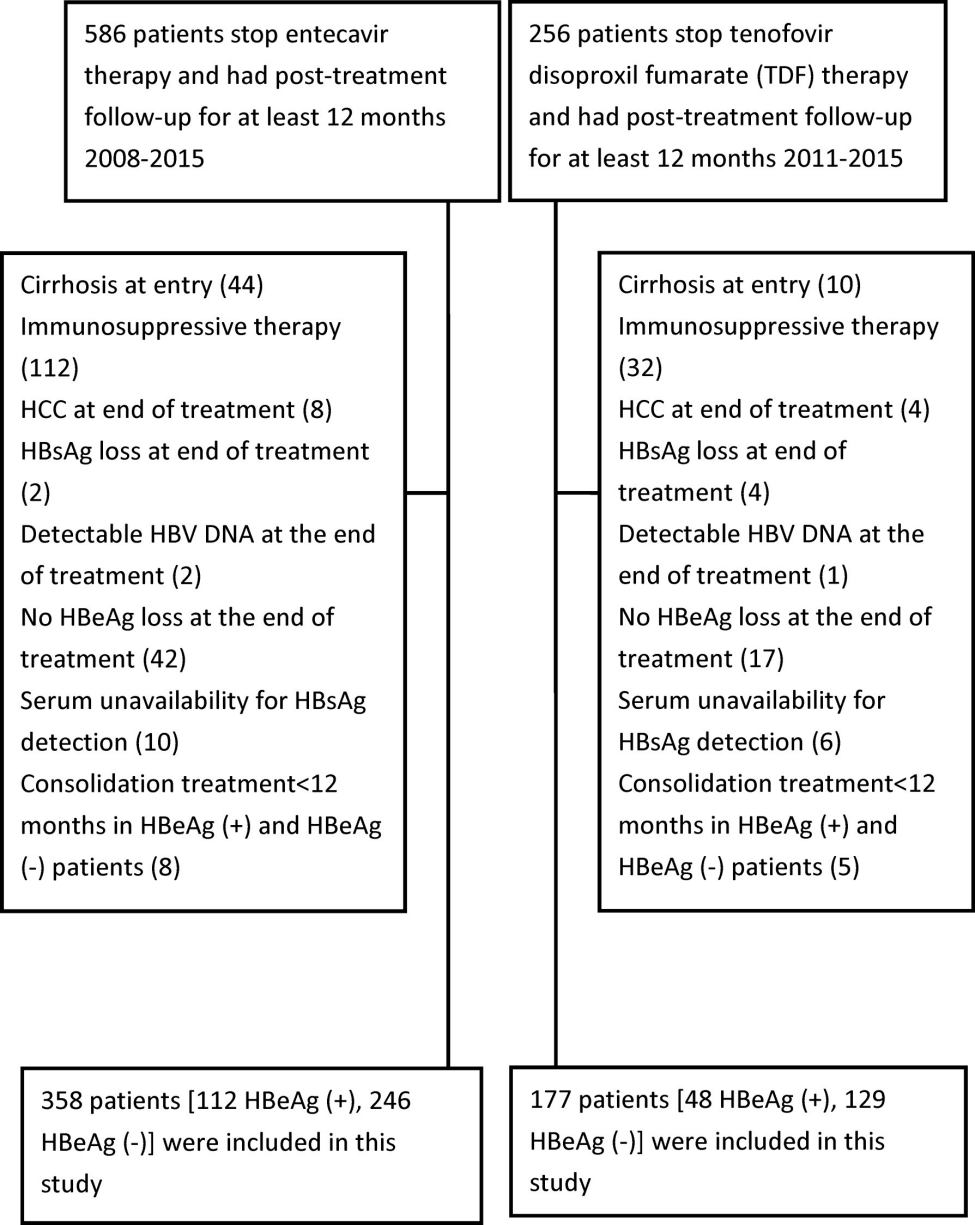
Flow chart of patients enrolled in this study.

Fig 1 shows the flowchart of patients enrolled in this prospectively retrospective cohort study. We enrolled 358 non-cirrhotic CHB patients who underwent ETV treatment between 2008 and 2015 (112 HBeAg-positive patients and 246 HBeAg-negative patients) and 177 non-cirrhotic CHB patients who underwent TDF treatment between 2011 and 2015 (48 HBeAg-positive patients and 129 HBeAg-negative patients). All of the patients were followed for at least 12 months after the discontinuation of NA therapy. Of the 358 ETV-treated patients, 301 were already included in our prior study to analyze HBsAg loss [10]. Non-cirrhosis was diagnosed based on biopsy (n = 20), a Fibrosis-4 (FIB-4) or liver stiffness measurement at baseline, or combined repeated ultrasound findings and without clinical features such as splenomegaly, thrombocytopenia, ascites, or gastroesophageal varices at baseline. In Taiwan, ETV and TDF has been reimbursed by National Health Plan for HBV treatment since august 2008 and June 2011, respectively. The selection of ETV or TDF was determined via a discussion between the patients and their physicians. All patients were tolerable and did not change drug during treatment.

Our patients satisfied the NA stopping criteria of the APASL 2012 [14]. During the study period, patients without liver cirrhosis were reimbursed for 3-year NA treatments by Taiwan's National Health Plan. When determining the treatment plan, the period of NA treatment was defined by the clinical physicians or the patients themselves. In order to avoid extra expenses, most patients (420/535; 78.5%) discontinued ETV or TDF therapy according to the criteria of Taiwan's National Health Plan.

Patients were excluded as described in detail previously [6,10,11]. The study was conducted in accordance with the Declaration of Helsinki of 1975. Written informed consent to participate in the study was obtained from all of the patients. All patients consented to providing serum for the purpose of checking HBV genotype, HBV DNA, and HBsAg levels. The Research Ethics Committees of Chang Gung Memorial Hospital (Kaohsinung, Taiwan) approved this study.

### Follow-up scheme

After discontinuation of either ETV or TDF therapy, the patients were followed as described in detail previously [11]. Serum samples from each patient were frozen and stored at -20°C at baseline, at month 12, and at the end of treatment for further use. Serum HBsAg quantification was investigated using the stored serum retrospectively.

### Definition of HBV relapse and criteria of retreatment

The treatment criteria required to initiate NA therapy according to Taiwan's National Health Plan are ALT ≥ 2× upper limit of normal (ULN) (40 U/L) plus HBV DNA > 20000 IU/mL in HBeAg-positive patients, and ALT elevation ≥ 2× ULN in two separate measurements obtained 3 months apart and HBV DNA ≥ 2000 IU/mL in HBeAg-negative patients without cirrhosis. Stopping criteria according to the APASL 2012 [14] include a combination of HBeAg seroconversion and undetectable HBV DNA levels for at least 12 months in HBeAg-positive patients. In HBeAg-negative patients, discontinuation is recommended when the patients have been treated for at least 2 years with undetectable HBV DNA documented on three separate occasions at least 6 months apart.

The definition of clinical relapse was ALT elevation > 2× ULN plus HBV DNA > 2000 IU/mL after discontinuation of NA therapy [14]. In HBeAg-positive patients, the definition of consolidation duration was the time elapsed from HBeAg seroconversion with undetectable HBV DNA until the end of the treatment regimen. In HBeAg-negative patients, the consolidation duration was calculated from the time of first undetectable HBV DNA until the end of the treatment regimen.

In non-cirrhotic patients, the criteria for retreatment according to Taiwan's National Health Plan are HBeAg reversion with an ALT elevation ≥ 2× ULN with HBV DNA ≥ 20000 IU/mL in HBeAg-positive patients and two measurements of ALT elevation ≥ 2× ULN obtained 3 months apart and HBV DNA ≥ 2000 IU/mL in HBeAg-negative patients. In patients with hepatic decompensation, the criteria for retreatment according to Taiwan's National Health Plan were total bilirubin ≥ 2 mg/dL or prolonged prothrombin time ≥ 3 seconds, regardless of HBV DNA levels.

According to the APASL guidelines of 2015 [3], hepatic decompensation was defined as total bilirubin > 3.5 mg/dL (2.5 times greater than the ULN (1.4 mg/dL)) and prolonged prothrombin time>3 seconds or INF >1.5.

### Serology and HBV genotyping

HBsAg, HBeAg, anti-hepatitis C virus antibodies, anti-hepatitis D virus antibodies, and HBV DNA and HBsAg levels were determined as described in detail previously in the literature [6,10,11].

HBV genotypes were determined using restriction fragment length polymorphism (RFLP) on the surface gene, as described previously in the literature [15]. Direct sequencing of surface genes was performed if the results of HBV genotype by RFLP were ambiguous or indicated a mixed infection. Compared with direct sequencing, the accuracy of HBV genotyping with this RFLP method exceeded 95%.

### Statistical analysis

Data are presented as means±standard deviation (SD), proportions, or median (range). Differences between the two of groups of patients were evaluated using the chi-squared test or Fisher’s exact test and the Student’s *t*-test for categorical and continuous variables, respectively. The cumulative incidence of clinical relapse and retreatment was estimated using the Kaplan-Meier method and compared using a log-rank test. Univariate and multivariate Cox proportional hazards regression models were used to identify factors associated with clinical relapse and meeting the criteria for retreatment. All variables with *P*<0.2 in the univariate analysis are required to allow into the multivariate analysis. Cox proportional hazards regression models with the forward method were used to determine independent factors, and variables with *P*<0.05 were required to stay in the models. All tests were two-sided, and *P* values less than 0.05 were considered to be statistically significant. Time-dependent receiver-operating characteristic (ROC) curve analysis was used to determine the cut-off values of HBsAg levels for predicting clinical relapse and retreatment within 5 years after discontinuation of either ETV or TDF treatment [16].

The propensity score (PS)-matching method was used to reduce the significant differences in clinical features between the ETV and TDF groups by creating a ratio of 1:1 to 1:2. After subgrouping by HBeAg status, the variables used in the model included age, gender, baseline ALT, HBV genotype, NA-naïve, baseline HBV DNA levels, treatment and consolidation duration, and HBsAg levels at baseline and end of treatment in the ETV and TDF groups. Caliper matching was performed on the propensity score (nearest available matching). Pairs (discontinuing and continuing groups) on the propensity score logit were matched to within a range of 0.2 SD.

## Results

### Clinical characteristics of the study population

A total of 535 patients were enrolled in the study (160 HBeAg-positive patients and 375 HBeAg-negative patients). The clinical features of the study population are listed in Table 1. A male gender and an HBV genotype B were predominant among the study population. The median duration from the end of treatment until retreatment was 42 weeks in HBeAg-positive patients and 64 weeks in HBeAg-negative patients. Compared with HBeAg-negative patients, HBeAg-positive patients were with younger, more often female and of HBV genotype C, characterized by a shorter consolidation duration, and more likely to exhibit higher levels of ALT, HBV DNA, and HBsAg at entry.

**Table 1:** Baseline characteristics of the study population.

Characteristics	HBeAg-positive patients n = 160	HBeAg-negative patients n = 375	*p* value
Age (years)	39.6±11.1	51.0±10.4	<0.001
Male gender	109 (68.1%)	306 (81.6%)	0.001
ALT (U/L)	470.5±490.9	312.6±442.6	0.001
Total bilirubin (mg/dL)	1.86±2.55	1.95±2.99	0.77
HBV DNA (log IU/mL)	6.90±1.43	5.82±1.57	<0.001
HBV genotype			
B	93 (58.1%)	298 (79.5%)	<0.001
C	67 (41.9%)	77 (20.5%)	
Treatment duration (weeks)	165.7±40.0	163.8±33.0	0.56
Consolidation duration (weeks)	100.6±39.1	131.8±37.6	<0.001
NA-naïve*	117 (73.1%)	274 (73.1%)	0.99
HBsAg at baseline (log IU/mL)	3.70±0.77	3.01±0.84	<0.001
HBsAg at the end of treatment (log IU/mL)	2.94±0.68	2.45±0.84	<0.001

ALT, alanine aminotransferase; HBV, hepatitis B virus, HBsAg, hepatitis B surface antigen; NA, nucleos(t)ide analogues.

*NA (nucleos(t)ide analogues)-naïve: no treatment history of NA before starting ETV or TDF therapy.

After stopping NA therapy, a total of 286 patients experienced clinical relapse during the follow-up period (78 in the HBeAg-positive group and 208 in the HBeAg-negative group). The median duration from the end of treatment until clinical relapse was 40 weeks (range: 7–411 weeks). In addition, a total of 234 patients received retreatment during off-therapy follow-up. The median duration from the end of treatment until retreatment was 57 weeks (range: 10–411 weeks). Of these patients, 65 were in the HBeAg-positive group and 169 were in the HBeAg-negative group. A total of 202 (86.3%) patients fulfilled the retreatment criteria of Taiwan's National Health Plan. Thirty-two (13.7%) patients received retreatment at their own expense under the patients' willingness.

The clinical characteristics of the patients at baseline and the end of treatment of ETV and TDF treatment are listed in Table 2. The TDF group was generally older, had higher baseline HBV DNA levels, a shorter treatment and consolidation duration, and a lower rate of NA-naïve status compared with patients in the ETV group. There were no significant differences in clinical characteristics at end of treatment between the two groups. To reduce the significant differences in baseline and on-treatment factors, the PS-matching method yielded 277 and 170 patients in the ETV and TDF groups, respectively. The baseline characteristics of both groups according to PS-matching method by HBeAg status are shown in S1 Table.

**Table 2:** Clinical features of baseline and the end of treatment for patients receiving entecavir (ETV) or tenofovir disoproxil fumarate (TDF) therapy.

Characteristics	ETV group n = 358	TDF group n = 177	*p* value
Starting time			
Age (years)	46.9±12.0	49.0±11.5	0.048
Male gender	275 (76.8%)	140 (79.1%)	0.55
ALT (U/L)	372.9±452.9	356.6±481.2	0.70
Total bilirubin (mg/dL)	1.60±2.26	1.96±3.69	0.31
HBeAg-positive status	112 (31.3%)	48 (27.1%)	0.32
HBV DNA (log IU/mL)	6.00±1.58	6.44±1.61	0.003
HBV genotype			
B	258 (72.1%)	133 (75.1%)	0.45
C	100 (27.9%)	44 (24.9%)	
Treatment duration (weeks)	166.59±41.51	159.84±15.36	0.007
Consolidation duration (weeks)	125.26±45.15	116.79±28.56	0.009
NA-naive	272 (76.0%)	119 (67.2%)	0.03
HBsAg at baseline (log IU/mL)	3.24±0.86	3.17±0.90	0.39
End of treatment			
Age (years)	50.07±11.97	52.01±11.44	0.068
ALT (U/L)	25.4±16.5	30.5±32.7	0.11
Total bilirubin (mg/dL)	0.99±0.38	1.01±0.96	0.25
HBV DNA <20 IU/mL	358 (100%)	177 (100%)	1
HBsAg at the end of treatment (log IU/mL)	2.63±0.82	2.52±0.83	0.16

ALT, alanine aminotransferase; HBV, hepatitis B virus, HBsAg, hepatitis B surface antigen; NA, nucleos(t)ide analogues.

### Incidence and predictors of clinical relapse and retreatment in HBeAg-positive CHB patients

**Fig 2:**
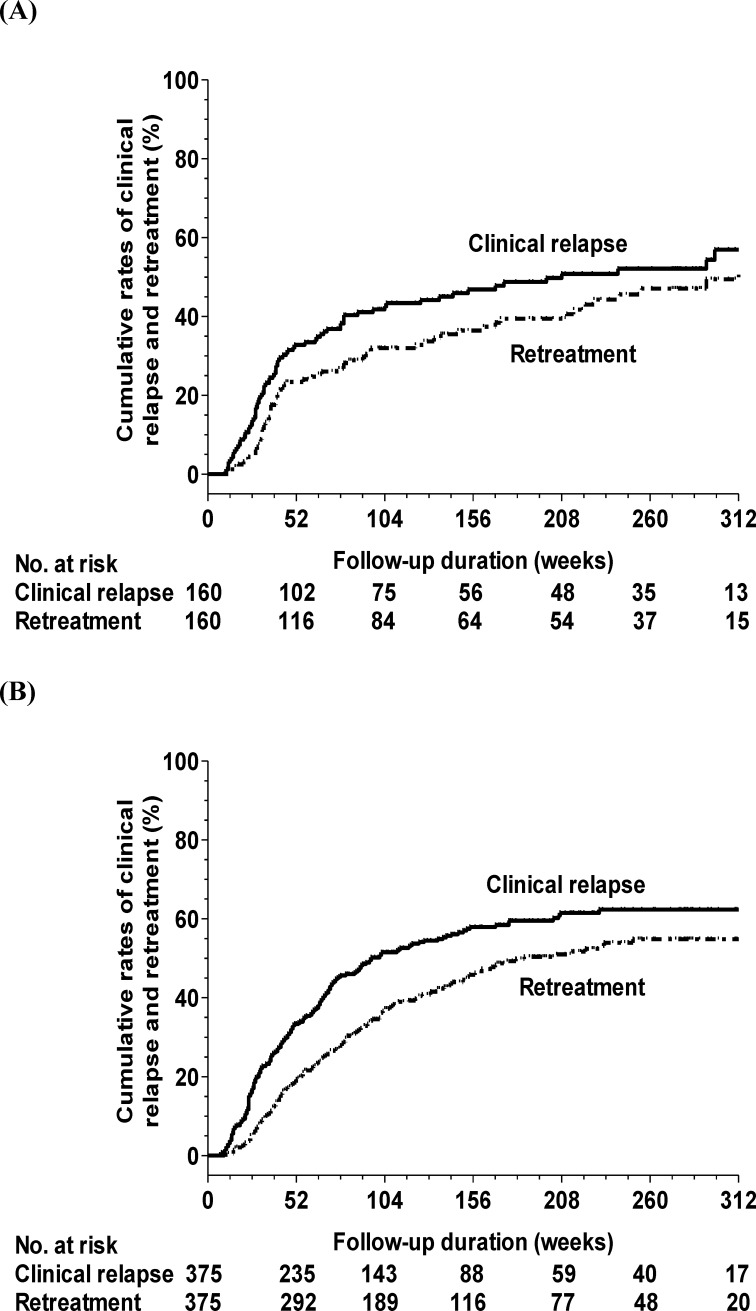
Cumulative incidences of clinical relapse and retreatment in (A) HBeAg-positive and (B) HBeAg-negative patients.

Among the 160 HBeAg-positive patients, the median duration from the end of treatment until clinical relapse was 37 weeks (range: 10–298 weeks). The cumulative incidences of clinical relapse at 1, 3, and 5 years were 32.8%, 46.8%, and 52.1%, respectively (Fig 2A). Baseline factors and HBsAg levels as Table 3 were included in the multivariate analysis. Multivariate Cox regression analysis revealed that the TDF group (Hazard ratio (HR): 2.69, 95% confidence interval (CI): 1.66–4.35, *P*<0.001), old age (increase per year, HR: 1.07, 95% CI: 1.05–1.10, *P*<0.001), male gender (HR: 2.93, 95% CI: 1.68–5.10, *P*<0.001), higher baseline HBsAg (increase per log IU/mL, HR: 1.88, 95% CI: 1.33–2.65, *P*<0.001), and higher end-of-treatment HBsAg levels (increase per log IU/mL, HR: 1.59, 95% CI: 1.12–2.27, *P*<0.001) were independent factors of clinical relapse. In the ETV group, the cumulative incidences of clinical relapse at 6, 12, 24, and 36 months were 5.4%, 24.2%, 33.8%, and 38.2%, respectively. In the TDF group, the cumulative rates of clinical relapse at 6, 12, 24, and 36 months were 33.3%, 53.4%, 63.9%, and 68.5%, respectively. Patients who discontinued TDF therapy had significantly higher clinical relapse rates than those who discontinued ETV therapy in all (*P* <0.001) and PS-matched patients (the cumulative rate at 36 months: ETV vs. TDF: 41.6% vs. 67.1%, *P* = 0.001).

**Table 3:** Factors predicting retreatment for HBeAg-positive patients.

Variables	Univariate analysis		Multivariate analysis	
	HR (95% CI)	*P* value	HR (95% CI)	*P* value
Age (per year)	1.044 (1.023–1.065)	<0.001	1.063 (1.036–1.091)	<0.001
Male gender	1.695 (0.952–3.019)	0.073	2.576 (1.403–4.729)	0.002
TDF vs. ETV	2.484 (1.449–4.259)	0.001	2.287 (1.328–3.939)	0.003
ALT (per U/mL)	1.000 (1.000–1.001)	0.218		
Total bilirubin (per mg/dL)	0.960 (0.863–1.067)	0.444		
HBV DNA (per log IU/mL) at baseline	1.155 (0.963–1.384)	0.120		
HBV genotype (C vs. B)	1.728 (1.061–2.815)	0.028		
NA-naïve	0.548 (0.329–0.912)	0.021		
HBsAg at baseline (per log IU/L)	1.369 (0.968–1.935)	0.076	1.815 (1.275–2.589)	0.001
HBsAg at month 12 of treatment (per log IU/L)	1.297 (0.854–1.970)	0.222		
HBsAg at the end of treatment (per log IU/L)	1.244 (0.865–1.788)	0.293	.	
HBsAg decline from baseline to end of treatment (per log IU/L)	1.135 (0.937–1.374)	0.196		
Treatment duration (per week)	1.001 (0.995–1.006)	0.802		
Consolidation duration (per week)	1.003 (0.998–1.009)	0.257		

ALT, alanine aminotransferase; CI, confidence interval; HR, hazard ratio; HBV, hepatitis B virus, HBsAg, hepatitis B surface antigen; NA, nucleos(t)ide analogues; TDF, tenofovir disoproxil fumarate

**Fig 3:**
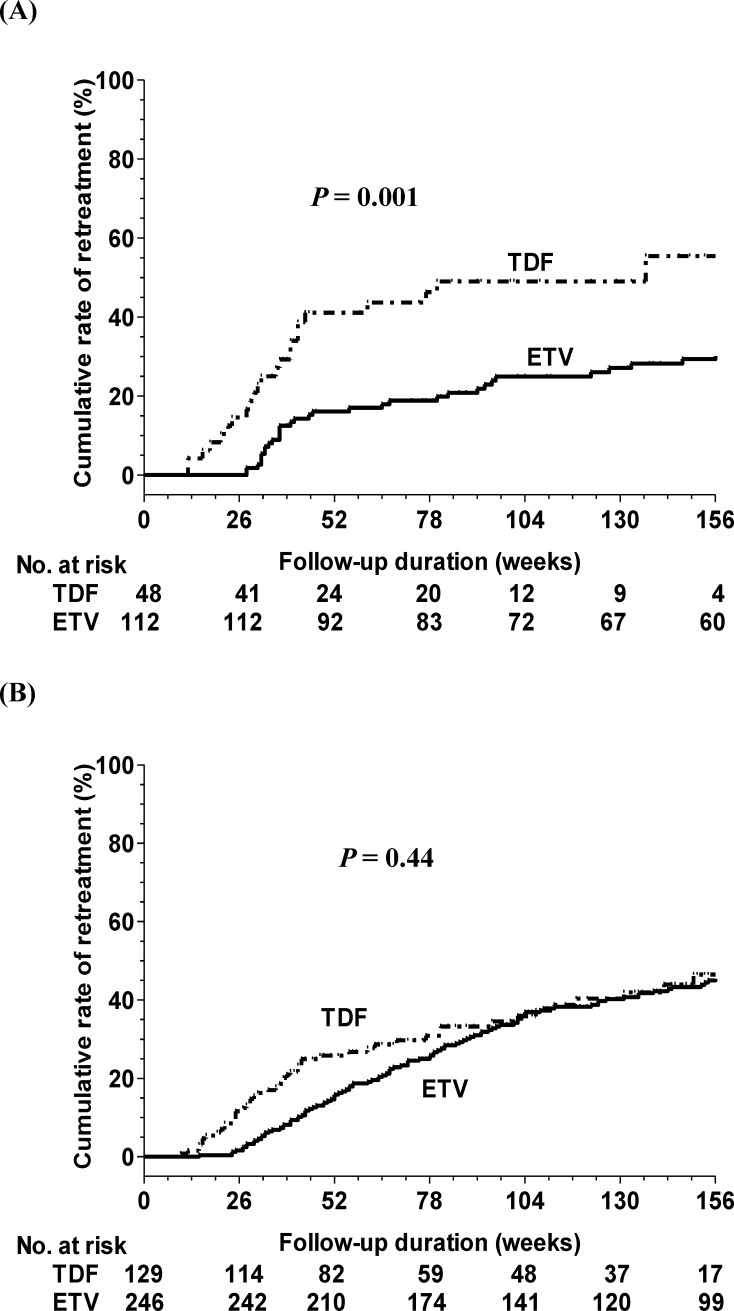
Comparison of the incidences of retreatment between entecavir (ETV) and tenofovir disoproxil fumarate (TDF) in (A) HBeAg-positive and (B) HBeAg-negative patients.

The median duration from the end of treatment until retreatment was 42 weeks (range: 12–316 weeks). The cumulative incidences of retreatment at 1, 3, and 5 years were 23.4%, 36.4%, and 47%, respectively (Fig 2A). Multivariate Cox regression analysis revealed that the TDF group, an older age, a male gender, and higher baseline HBsAg levels were independent factors of retreatment (Table 3). In the ETV group, the cumulative rates of retreatment at 6, 12, 24, and 36 months were 1.8%, 16.1%, 24.9%, and 29.4%, respectively. In the TDF group, the cumulative rates of retreatment at 6, 12, 24, and 36 months were 14.6%, 41.1%, 49%, and 55.4%, respectively. Patients who discontinued TDF therapy had significantly higher retreatment rates than patients who discontinued ETV therapy in all (*P* = 0.001) (Fig 3A) and PS-matched patients (the cumulative rate at 36 months: ETV vs. TDF: 32.2% vs. 53.3%, *P* = 0.011).

### Incidence and predictors of clinical relapse and retreatment in HBeAg-negative CHB patients

Among the 375 HBeAg-negative patients, the median duration from end of treatment until clinical relapse was 43 weeks (range: 7–411 weeks). The cumulative incidences of clinical relapse at 1, 3, and 5 years were 33.1%, 57.6%, and 62%, respectively (Fig 2B). Baseline factors and HBsAg levels as Table 4 were included in the multivariate analysis. Multivariate Cox regression analysis revealed that the TDF group (HR: 1.44, 95% CI: 1.07–1.93, *P* = 0.017), an oldage (increase per year, HR: 1.03, 95% CI: 1.01–1.14, *P*<0.001), HBV genotype B (HR: 2.14, 95% CI: 1.44–3.17, *P*<0.001), and higher end-of-treatment HBsAg levels (increase per log IU/mL, HR: 2.02, 95% CI: 1.61–2.52, *P*<0.001) were independent factors of clinical relapse. In the ETV group, the cumulative rates of clinical relapse at 6, 12, 24, and 36 months were 11%, 30.6%, 48.7%, and 55.5%, respectively. In the TDF group, the cumulative rates of clinical relapse at 6, 12, 24, and 36 months were 28%, 38.4%, 56.1%, and 61.8%, respectively. Patients who discontinued TDF therapy had a borderline significantly higher clinical relapse rate than patients who discontinued ETV therapy in all (*P* = 0.05) and PS-matched patients (the cumulative rate at 36 months: ETV vs. TDF: 56.1% vs. 62.4%, *P* = 0.08).

**Table 4:** Factors predicting retreatment for HBeAg-negative patients.

Variables	Univariate analysis		Multivariate analysis	
	HR (95% CI)	*P* value	HR (95% CI)	*P* value
Age (per year)	1.022 (1.007–1.037)	0.003	1.033 (1.018–1.049)	<0.001
Male gender	1.225 (0.816–1.838)	0.328		
TDF vs. ETV	1.143 (0.813–1.607)	0.441		
ALT (per U/mL)	1.000 (1.000–1.001)	0.189		
Total bilirubin (per mg/dL)	0.986 (0.930–1.045)	0.629		
HBV DNA (per log IU/mL) at baseline	1.232 (1.109–1.369)	<0.001		
HBV genotype (C vs. B)	0.581 (0.384–0.877)	0.010	0.457 (0.295–0.708)	<0.001
NA-naïve	0.936 (0.663–1.320)	0.704		
HBsAg at baseline (per log IU/L)	1.437 (1.199–1.721)	<0.001	1.322 (1.060–1.648)	0.013
HBsAg at month 12 of treatment (per log IU/L)	1.440 (1.163–1.178)	0.001		
HBsAg at the end of treatment (per log IU/L)	1.422 (1.174–1.721)	<0.001	1.663 (1.258–2.197)	<0.001
HBsAg decline from baseline to end of treatment (per log IU/L)	1.004 (0.886–1.139)	0.945		
Treatment duration (per week)	0.998 (0.994–1.003)	0.495		
Consolidation duration (per week)	0.995 (0.990–0.999)	0.022		

ALT, alanine aminotransferase; CI, confidence interval; HR, hazard ratio; HBV, hepatitis B virus, HBsAg: hepatitis B surface antigen; NA, nucleoside analogues; TDF, tenofovir disoproxil fumarate

The median duration from end of treatment until retreatment was 98 weeks (range: 10–472 weeks). The cumulative incidences of retreatment at 1, 3, and 5 years were 19.3%, 45.8%, and 54.8%, respectively (Fig 2B). As listed in Table 4, multivariate Cox regression analysis revealed that an old age, HBV genotype B status, and higher HBsAg levels at baseline and at the end of treatment were independent factors of retreatment. In the ETV group, the cumulative rates of retreatment at 6, 12, 24, and 36 months were 1.6%, 15.9%, 36.8%, and 45%, respectively. In the TDF group, the cumulative rates of retreatment at 6, 12, 24, and 36 months were 11.6%, 25.8%, 35.8%, and 46.5%, respectively. There was no significant difference in terms of the retreatment rate between the TDF and ETV groups in all (*P* = 0.44) (Fig 3B) and PS-matched patients (the cumulative rate at 36 months: ETV vs. TDF: 48.2% vs. 47.9%, *P* = 0.69).

### Optimal cut-off-point of serum HBsAg levels for predicting clinical relapse and retreatment

**Fig 4:**
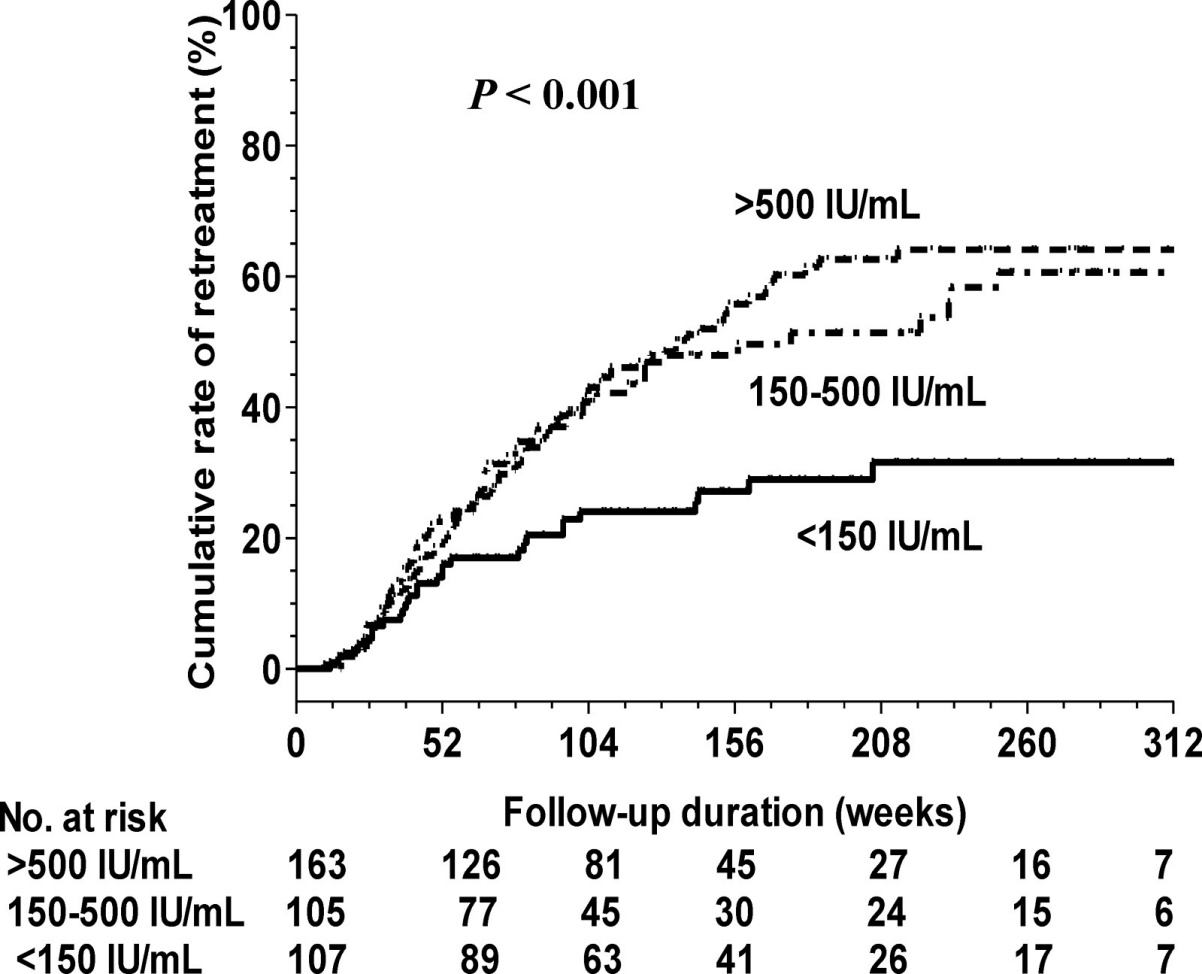
Cumulative incidences of retreatment according to end-of treatment HBsAg levels in HBeAg-negative patients.

Because end-of-treatment HBsAg level was an independent predictor of clinical relapse and retreatment in HBeAg-negative patients, we attempted to determine the best cut-off value for predicting clinical relapse and retreatment. Time-dependent ROC analysis revealed that the best cut-off value of HBsAg for predicting clinical relapse was 138.9 IU/mL (area under the ROC curve: 0.639; sensitivity: 82.8%; specificity: 54.8%) and retreatment was 169.28 IU/mL (area under the ROC curve: 0.629; sensitivity: 81.4%; specificity: 55.2%) within 5 years after discontinuation of either ETV or TDF treatment. We adopted an HBsAg level of 150 IU/mL at the end of treatment as an optimal value for predicting retreatment in HBeAg-negative patients (*P* < 0.001). The 5-year cumulative retreatment rates were 31.6%, 60.6%, and 64.1% for patients who achieved end-of-treatment HBsAg levels of <150, 150–500, and >500 IU/mL, respectively (Fig 4).

### Highest ALT levels and hepatic decompensation upon retreatment

Among the 234 patients who received retreatment, 128, 66, and 12 patients experienced the highest ALT levels (>200, >400, and >1000 U/L upon retreatment, respectively). Total bilirubin ≥ 2 and ≥ 3 mg/dL upon retreatment developed in 44 (18.8%) and 10 (4.3%) patients, respectively, and prolonged PT ≥3 seconds upon retreatment developed in 12 (5.1%) patients (Table 5). Therefore, 18.8% patients who received retreatment fulfilled the definition of retreatment of hepatic decompensation according to Taiwan's National Health Plan. There was no significant factor at baseline and end of treatment between patients with and without hepatic decompensation upon retreatment (Table 5)

**Table 5:** Clinical characteristics of patients with or without hepatic decompensation upon retreatment according to the criteria of Taiwan's National Health Plan.

Characteristics	Patient without hepatic decompensation n = 190	Patient with hepatic decompensation n = 44	*p* value
Starting time			
Age (years)	49.3±11.3	50.4±9.8	0.58
Male gender	150 (78.9%)	40 (90.9%)	0.086
ALT (U/L)	404.6±539.9	454.8±548.6	0.58
Total bilirubin (mg/dL)	1.72±2.66	2.20±2.15	0.26
HBeAg-positive status	54 (28.4%)	11 (25%)	0.65
HBV DNA (log IU/mL)	6.35±1.36	6.51±1.58	0.50
HBV genotype			
B	139 (73.2%)	33 (75%)	0.80
C	51 (26.8%)	11 (25%)	
FIB-4	3.16±0.83	3.54±2.64	0.17
Treatment duration (weeks)	163.6±37.1	161.2±20.6	0.69
Consolidation duration (weeks)	121.9±39.2	118.3±29.9	0.57
ETV vs. TDF	137 vs. 53	26 vs. 18	0.091
NA-naive	140 (73.7%)	28 (63.6%)	0.18
HBsAg at baseline (log IU/mL)	3.26±0.78	3.45±0.71	0.47
End of treatment			
Age (years)	52.59±11.42	4.32±10.54	0.36
ALT (U/L)	28.5±21.1	26.8±13.0	0.61
Total bilirubin (mg/dL)	0.98±1.54	1.20±0.51	0.39
HBV DNA <20 IU/mL	190 (100%)	44 (100%)	1
FIB-4	1.67±0.90	1.92±1.01	0.17
HBsAg at the end of treatment (log IU/mL)	2.80±0.56	2.67±0.55	0.15

ALT, alanine aminotransferase; FIB-4: fibrosis 4; ETV, entecavir; HBsAg, hepatitis B surface antigen; PT, prothrombin time; TDF, tenofovir disoproxil fumarate.

In addition, 7 (3%) patients who received retreatment fulfilled the definition of hepatic decompensation according to the 2015 APASL guidelines [3]. Univariate and multivariate analysis revealed that only higher HBV DNA levels at baseline (Odds ratio (OR): 2.16, 95% CI: 1.02–4.58, *P* = 0.043) were a significant factor of hepatic decompensation according to the 2015 APASL guidelines after adjusting for all baseline and end-of-treatment factors. Three patients experienced clinical events of decompensation (2 ascites, 1 hepatic encephalopathy). The time to develop clinical events of decompensation was 15, 40, and 91 weeks after discontinuation of NA therapy. One patient died after timely retreatment.

### Clinical outcomes in patients with clinical relapse without retreatment

A total of 64 patients with clinical relapse did not receive retreatment. These patients did not meet the criteria for retreatment according to Taiwan's National Health Plan. Of these patients, 17 (26.6%) achieved sustained viral remission (HBV DNA ≤ 2000 IU/mL) and 26 (40.6%) did not experience clinical relapse (but HBV DNA >2000 IU/mL) from the follow up until the last visit after their clinical relapse. Twenty-eight experienced persistent ALT normalization (≤ 40 U/L) and 4 developed HBsAg loss after clinical relapse.

## Discussion

Our large-scale study revealed that 5-year cumulative retreatment rates were 47% and 54.8%, respectively, in HBeAg-positive and HBeAg-negative patients based on the retreatment criteria of Taiwan's National Health Plan. The retreatment rate represented the rate of persistent hepatitis flare (including ALT and HBV DNA) after discontinuation of NA therapy. In our study, the median duration from the end of treatment until clinical relapse and retreatment was 40 and 57 weeks, respectively. The definitions of retreatment may have a significant impact on the probability of retreatment. According to the retreatment criteria of Taiwan's National Health Plan, patients could be retreated if two measurements of ALT elevation ≥ 2× ULN obtained 3 months apart and HBV DNA ≥ 2000 IU/mL in HBeAg-negative status after discontinuation of NA therapy. Thus, the criteria might be the main reason for the difference of 17 weeks between clinical relapse and retreatment.

One recent study in Taiwan reported that 40 out of 100 patients (40%) (including HBeAg-positive and HBeAg-negative patients at entry) received retreatment for 35 weeks of follow-up after stopping either ETV or TDF therapy [12]. A study in Korea showed that 12 of 45 (26.6%) HBeAg-positive patients and 27 of 68 (39.7%) HBeAg-negative patients received retreatment after stopping either ETV or lamivudine therapy (the criteria of retreatment after HBV relapse were not defined) [17]. A recent study in Greece that enrolled 57 non-cirrhotic, HBeAg-negative CHB patients who discontinued effective ≥ 4-year ETV or TDF therapy showed that the cumulative rate of retreatment was 18% and 26% at 3 and 12 months, respectively [18]. The retreatment criteria of the Greece study were ALT >10× ULN or ALT >5× ULN and total bilirubin >2 mg/dL at the same visit, ALT >3× ULN and HBV DNA >100,000 IU/mL at three sequential visits. A collaborative Greek and Taiwanese study enrolling 130 non-cirrhotic HBeAg-negative patients showed that cumulative retreatment rates were 22% and 40% 12 and 24 months, respectively, after stopping NA therapy according to the retreatment criteria of Greece and Taiwan [19].

The effects of baseline factors in terms of predicting retreatment after stopping NA therapy have not been well established. In our study, multivariate Cox regression analysis revealed that the status of discontinuing TDF therapy, an older age, a male gender, and higher HBsAg levels at baseline were independent factors of retreatment in HBeAg-positive patients; an HBV genotype B, an older age, and higher HBsAg levels at baseline and end of treatment were independent factors of retreatment in HBeAg-negative patients. At the end of treatment, an HBsAg level of 150 IU/mL was an optimal value for predicting retreatment in HBeAg-negative patients. A collaborative Greek and Taiwanese study demonstrated that the probability of retreatment was significantly associated with lower platelets at NA onset. An older age and the use of TDF as the last NA agent had increasing trends on future retreatment [19]. However, none of the above factors was significantly associated with retreatment in the multivariate regression [19].

Our study found that the predictors of clinical relapse were similar with retreatment. Recent studies have reported that the timing of HBV relapse after discontinuation of TDF was much earlier than that after discontinuation of ETV in CHB patients [7,11,12]. Until now, the mechanism of different relapse patterns between ETV and TDF discontinuation has remained unclear. HBV viral replication can resume from residual covalently closed circular DNA (cccDNA) after discontinuation of NA therapy. Therefore, we speculated that ETV might suppress cccDNA to a lower level and that more time might be required to resume HBV replication after the cessation of therapy. However, this hypothesis requires further study. In our investigation, HBeAg-positive patients who discontinued TDF therapy had significantly higher retreatment rates than HBeAg-positive patients who discontinued ETV therapy; the same situation did not persist for HBeAg-negative patients. This finding may be explained be noting that HBeAg-positive patients in the TDF group had significantly higher clinical relapse rates than HBeAg-positive patients in the ETV group.

Severe acute exacerbation with hepatic decompensation after stopping NA therapy and the effect of ALT elevation upon retreatment are important issues. Our study showed that 54.7%, 28.2%, and 5.1% of patients experienced the highest ALT levels of >200, >400, and >1000 U/L upon retreatment, respectively. A total of 18.8% and 3% patients who received retreatment fulfilled the definitions of hepatic decompensation according to Taiwan's National Health Plan and the 2015 APASL guidelines [3], respectively. Because ALT flare with hepatic decompensation is hard to avoid during off-NA-therapy follow-up, close monitoring after discontinuing NA therapy is necessary for timely retreatment to reduce the risk of hepatic decompensation and mortality. However, the clinical characteristics were similar between patients with and without hepatic decompensation upon retreatment according to criteria of Taiwan's National Health Plan.

A recent study showed that patients with clinical relapse but without retreatment had a higher incidence of HBsAg clearance than patients who received retreatment [13]. In our study, among the 64 patients with clinical relapse but without retreatment, 43 (67.2%) patients did not experience clinical relapse during the follow-up until their last visit after clinical relapse. Among these 43 patients, 17 achieved sustained viral remission (4 developed HBsAg seroclearance). These findings may support the notion that patients with transient ALT elevation and a subsequent decrease in viral load might have better immune control, which may increase HBsAg seroclearance rate. Therefore, close monitoring instead of immediate retreatment is suggested for patients who experience clinical relapse without hepatic decompensation. A recent study suggested that HBsAg levels decrease in patients with hepatitis flaring and that retreatment can be withheld or is not necessary. In contrast, retreatment should be considered for patients with elevated serum HBsAg and ALT levels [20]. Additional studies are necessary to differentiate the effects of ALT flaring on future outcomes to determine the best time for retreatment.

Our study has some limitations. First, this investigation was a retrospective and single-center study. Additional multicenter and prospective studies are necessary to confirm our results. Second, our study included only Asian participants, among whom HBV genotypes B and C are predominant [15]. Furthermore, the criteria of retreatment were those of Taiwan's National Health Plan. Therefore, the incidence and predictors of retreatment might differ from other HBV genotypes and retreatment criteria. Third, we enrolled a small proportion of patients undergoing retreatment at their own expense, which might have increased the rate of retreatment. Forth, the diagnosis of non-cirrhosis for some patients was based on clinical criteria and ultrasound. Ultrasonography is more likely to result in an underestimation of early stages of cirrhosis.

## Conclusions

The 5-year cumulative incidences of retreatment were 47% in HBeAg-positive patients and 54.8% in HBeAg-negative patients after discontinuation of either ETV or TDF therapy according to the criteria of retreatment of Taiwan's National Health Plan. HBeAg-positive patients who discontinued TDF therapy had significantly higher retreatment rates than HBeAg-positive patients who discontinued ETV therapy. Close monitoring for timely retreatment is necessary to reduce the risk of hepatic failure or mortality after discontinuation of NA therapy.

## Supporting information

S1 Data Raw data. (XLSX)

S1 Table Baseline characteristics of study population by propensity score matching method. (DOCX)

## References

[1] Weinbaum CM, Mast EE, Ward JW. Recommendations for Identification and Public Health Management of Persons with Chronic Hepatitis B Virus Infection. Hepatology 2009;49:535–544. 10.1002/hep.22882 19399812

[2] Liaw YF, Chu CM. Hepatitis B virus infection. Lancet 2009;373:582–592. 10.1016/S0140-6736(09)60207-5 19217993

[3] Sarin SK, Kumar M, Lau GK, Abbas Z, Chan HL, Chen CJ, et al Asian-Pacific clinical practice guidelines on the management of hepatitis B: a 2015 update. Hepatol Int. 2016;10:1–98. 10.1007/s12072-015-9675-4 PMC4722087 26563120

[4] European Association for the Study of the Liver. EASL 2017 Clinical Practice Guidelines on the management of hepatitis B virus infection. J Hepatol 2017;67:370–398. 10.1016/j.jhep.2017.03.021 28427875

[5] Terrault NA, Bzowej NH, Chang KM, Hwang JP, Jonas MM, Murad MH. AASLD guidelines for treatment of chronic hepatitis B. Hepatology. 2016;63:261–283. 10.1002/hep.28156 26566064 PMC5987259

[6] Chen CH, Hung CH, Hu TH, Wang JH, Lu SN, Su PF, et al Association between level of hepatitis B surface antigen and relapse after entecavir therapy for chronic HBV infection. Clin Gastroenterol Hepatol 2015;13:1984–1992. 10.1016/j.cgh.2015.06.002 26073492

[7] Jeng WJ, Chen YC, Sheen IS, Lin CL, Hu TH, Chien RN, et al Clinical relapse after cessation of tenofovir therapy in hepatitis B e antigen-negative patients. Clin Gastroenterol Hepatol 2016;14:1813–1820. 10.1016/j.cgh.2016.07.002 27404969

[8] Jeng WJ, Sheen IS, Chen YC, Hsu CW, Chien RN, Chu CM, et al Off-therapy durability of response to entecavir therapy in hepatitis B e antigen-negative chronic hepatitis B patients. Hepatology 2013;58:1888–1896. 10.1002/hep.26549 23744454

[9] Chen CH, Hsu YC, Lu SN, Hung CH, Wang JH, Lee CM, et al The incidence and predictors of HBV relapse after cessation of tenofovir therapy in chronic hepatitis B patients. J Viral Hepat 2018;25:590–597. 10.1111/jvh.12851 29274189

[10] Chen CH, Hung CH, Wang JH, Lu SN, Hu TH, Lee CM. Long-term incidence and predictors of hepatitis B surface antigen loss after discontinuing nucleoside analogues in noncirrhotic chronic hepatitis B patients. Clin Microbiol Infect 2018;24:997–1003. 10.1016/j.cmi.2017.12.013 29288020

[11] Kuo MT, Hu TH, Hung CH, Wang JH, Sheng-Nan Lu SN, Tsai KL, et al Comparison of hepatitis B virus relapse rates between chronic hepatitis B patients who discontinue entecavir and tenofovir treatment. Aliment Pharmacol Ther 2019;49:218–228. 10.1111/apt.15053 30484881

[12] Su TH, Yang HC, Tseng TC, Liu CJ, Kao JH. Distinct relapse rates and risk predictors after Discontinuing tenofovir and entecavir therapy. J Infect Dis 2018;21:1193–1201. 10.1093/infdis/jix690 29300980

[13] Jeng WJ, Chen YC, Chien RN, Sheen IS, Liaw YF. Incidence and predictors of hepatitis B surface antigen seroclearance after cessation of nucleos(t)ide analogue therapy in hepatitis B e antigen-negative chronic hepatitis B. Hepatology 2018;68:425–434. 10.1002/hep.29640 29108132

[14] Liaw YF, Kao JH, Piratvisuth T, Chan HL, Chien RN, Liu CJ, et al Asian-Pacific consensus statement on the management of chronic hepatitis B: a 2012 update. Hepatol Int 2012;6:531–561. 10.1007/s12072-012-9365-4 26201469

[15] Lee CM, Chen CH, Lu SN, Tung HD, Chou WJ, Wang JH, et al Prevalence and clinical implications of hepatitis B virus genotypes in southern Taiwan. Scand J Gastroenterol 2003;38:95–101. 10.1080/00365520310000500 12608471

[16] Heagerty PJ, Lumley T, Pepe MS. Time-dependent ROC curves for censored survival data and a diagnostic marker. Biometrics 2000;56:337–344. 10877287 10.1111/j.0006-341x.2000.00337.x

[17] Xu WX, Zhang Q, Zhu X, Lin CS, Chen YM, Deng H1, et al 48-Week outcome after cessation of nucleos(t)ide analogue treatment in chronic hepatitis B patient and the associated factors with relapse. Can J Gastroenterol Hepatol 2018 5 10;2018:1817680 10.1155/2018/1817680 29862225 PMC5971349

[18] Papatheodoridis GV, Rigopoulou EI, Papatheodoridi M, Zachou K, Xourafas V, Gatselis N, et al DARING-B: discontinuation of effective entecavir or tenofovir disoproxil fumarate long-term therapy before HBsAg loss in non-cirrhotic HBeAg-negative chronic hepatitis B. Antivir Ther 2018, in press. 10.3851/IMP3256 30044765

[19] Papatheodoridis GV, Manolakopoulos S, Su TH, Siakavellas S, Liu CJ, Kourikou A, et al Significance of definitions of relapse after discontinuation of oral antivirals in HBeAg-negative chronic hepatitis B. Hepatology 2018;68:415–424. 10.1002/hep.29497 28859219

[20] Liaw YF, Jeng WJ, Chang ML. HBsAg kinetics in retreatment decision for off-therapy hepatitis B flare in HBeAg-negative patients. Gastroenterology. 2018;154:2280–2281. 10.1053/j.gastro.2018.03.066 29746811

