# Supplementary material for: Incidence and predictors of retreatment in chronic hepatitis B patients after discontinuation of entecavir or tenofovir treatment
Source: PLoS One. 2019 Oct 4;14(10):e0222221. doi: 10.1371/journal.pone.0222221 (PMC6777800; doi:10.1371/journal.pone.0222221)
Supplement: S1 Table — (DOCX) [file pone.0222221.s002.docx]

S**upplementary Table 1.** Baseline characteristics of study population by propensity score matching method

|  | HBeAg-positive patients | | | HBeAg-negative patients | | |
| --- | --- | --- | --- | --- | --- | --- |
|  | ETV group  n=83 | TDF group  n=46 | *P* value | ETV group  n=194 | TDF group  n=124 | *P* value |
| Age (years) | 40.5±11.8 | 41.7±10.3 | 0.84 | 51.3±10.1 | 51.8±10.8 | 0.70 |
| Sex (male: female) | 53:30 | 32:14 | 0.51 | 157:37 | 101:23 | 0.91 |
| ALT (U/L) | 424.9±428.3 | 422.4±558.5 | 0.98 | 314.6±442.1 | 314.1±430.8 | 0.99 |
| Total bilirubin (mg/dL) | 1.84±2.91 | 1.32±0.85 | 0.24 | 1.93±3.01 | 1.80±3.02 | 0.71 |
| HBV DNA (log IU/mL) | 7.16±1.19 | 7.15±1.41 | 0.99 | 5.96±1.38 | 6.13±1.63 | 0.31 |
| HBV genotype  B  C | 52 (62.6%)  31 (37.4%) | 28 (60.9%)  18 (39.1%) | 0.84 | 155 (79.9%)  39 (20.1%) | 102 (82.3%)  22 (17.7%) | 0.60 |
| Treatment duration (weeks) | 164.6±42.3 | 163.3±22.2 | 0.86 | 161.7±30.2 | 159.0±11.4 | 0.33 |
| Consolidation duration (weeks) | 97.9±37.4 | 98.6±32.6 | 0.92 | 129.7±33.6 | 124.9±22.3 | 0.15 |
| NA-naive | 61 (73.5%) | 31 (67.4%) | 0.46 | 143 (73.7%) | 87 (70.2%) | 0.49 |
| HBsAg at baseline  (log IU/mL) | 3.72±0.77 | 3.71±0.77 | 0.91 | 3.02±0.82 | 2.99±0.87 | 0.75 |
| HBsAg at the end of treatment (log IU/mL) | 2.96±0.63 | 2.98±0.67 | 0.83 | 2.47±0.80 | 2.38±0.82 | 0.34 |

ALT, alanine aminotransferase; ETV, entecavir; HBV, hepatitis B virus, HBsAg, hepatitis B surface antigen; HBeAg, hepatitis B e antigen; NA, nucleoside analogues; TDF, tenofovir disoproxil fumarate.
